# Genome-wide analysis of histone acetyltransferase and histone deacetylase families and their expression in fruit development and ripening stage of pepper (*Capsicum annuum*)

**DOI:** 10.3389/fpls.2022.971230

**Published:** 2022-09-07

**Authors:** Yutong Cai, Mengwei Xu, Jiarong Liu, Haiyue Zeng, Jiali Song, Binmei Sun, Siqi Chen, Qihui Deng, Jianjun Lei, Bihao Cao, Changming Chen, Muxi Chen, Kunhao Chen, Guoju Chen, Zhangsheng Zhu

**Affiliations:** ^1^Key Laboratory of Biology and Germplasm Enhancement of Horticultural Crops in South China, Ministry of Agriculture and Rural Areas, College of Horticulture, South China Agricultural University, Guangzhou, China; ^2^Peking University Institute of Advanced Agricultural Sciences, Weifang, China; ^3^School of Advanced Agricultural Sciences, Peking University, Beijing, China; ^4^Guangdong Helinong Seeds Co., Ltd., Shantou, China

**Keywords:** pepper, fruit, development, ripening, HAT and HDAC, histone modification

## Abstract

The fruit development and ripening process involve a series of changes regulated by fine-tune gene expression at the transcriptional level. Acetylation levels of histones on lysine residues are dynamically regulated by histone acetyltransferases (HATs) and histone deacetylases (HDACs), which play an essential role in the control of gene expression. However, their role in regulating fruit development and ripening process, especially in pepper (*Capsicum annuum*), a typical non-climacteric fruit, remains to understand. Herein, we performed genome-wide analyses of the HDAC and HAT family in the pepper, including phylogenetic analysis, gene structure, encoding protein conserved domain, and expression assays. A total of 30 HAT and 15 HDAC were identified from the pepper genome and the number of gene differentiation among species. The sequence and phylogenetic analysis of CaHDACs and CaHATs compared with other plant HDAC and HAT proteins revealed gene conserved and potential genus-specialized genes. Furthermore, fruit developmental trajectory expression profiles showed that *CaHDAC* and *CaHAT* genes were differentially expressed, suggesting that some are functionally divergent. The integrative analysis allowed us to propose CaHDAC and CaHAT candidates to be regulating fruit development and ripening-related phytohormone metabolism and signaling, which also accompanied capsaicinoid and carotenoid biosynthesis. This study provides new insights into the role of histone modification mediate development and ripening in non-climacteric fruits.

## Introduction

The dynamics of chromatin structure regulate DNA accessibility and DNA-templated processes and affect various biological processes of eukaryotes. The nucleosome is the basic unit of chromatin, and it compacts DNA nearly sevenfold, with about 146 bp of DNA wrapped around a histone octamer (Xu et al., 2015). The histone octamer is composed of two copies of H2A, H2B, H3, and H4 histone proteins (Arents et al., 1991). The unstructured amino-terminal tails and the globular domains determined that histones can be modified by dynamic post-translational modifications, including acetylation, methylation, ubiquitination, SUMOylation, phosphorylation, and less commonly by citrullination and ADP-ribosylation (Bannister and Kouzarides, 2011). Among these, histone acetylation is one of the well-characterized modifications; it has been shown that the acetylation of lysines is highly dynamic and regulated by the opposing action of two families of enzymes, histone acetyltransferases (HATs) and histone deacetylases (HDACs) (Stoll et al., 2018).

HATs and HDACs underlie a mechanism for reversibly modulating chromatin structure and transcriptional regulation (Bannister and Kouzarides, 2011). In plants, HATs have been distinctly divided into four groups, including (1) HAG for HATs of the GNAT (GCN5-related N-terminal acetyltransferases) superfamily; (2) HAM for HATs of the MYST superfamily; (3) HAC for HATs of the CREB-binding protein (CBP) family; (4) HAF for HATs of the TATA-binding protein-associated factor (TAFII250) family (Wang et al., 2014; Li et al., 2022). In parallel, plant HDACs have been classified into three families, including RPD3/HDA1 superfamily (HDA), Silent Information Regulator 2 (SRT), and HD2 (HDT) families (Chen X. et al., 2020). It was reported that changes in the acetylation level are associated with changes in gene expression by different endogenous and exogenous factors (Shen et al., 2015). HATs promote histone acetylation, leading to the generation of loose chromatin structures, facilitating the accessibility of promoters to components of the transcription machinery, and thus leading to transcriptional activation (Bannister and Kouzarides, 2011). Recently, the finding was that coactivator proteins are associated with HATs (Servet et al., 2010). HATs have been reported in the regulations of developmental transitions, integrations of hormone signals, and responses to environmental cues (Loidl, 2004). In contrast, the action of HDACs facilitating histone deacetylation is believed to be associated with a less accessible chromatin conformation (Yun et al., 2011), and corepressor proteins always form the complex with HDACs, resulting in gene silencing (Deng et al., 2022; Hu et al., 2022);. Studies have revealed they play key roles in regulating plant vegetative and reproductive development, stress responses, gene silencing, as well as cell death and cycle (Shen et al., 2015). These studies suggest that HATs and HDACs are essential for transcription regulation.

Fruit development comprises fruit set initiation, growth, maturation, and ripening; phytohormones auxins, gibberellins (GAs), cytokinins, abscisic acid (ABA), and ethylene have been implicated at various stages of the fruit growth cycle (White, 2002). Fruit development consists of cell division and expansion, the former shown to be influenced by auxin signaling, and cell expansion is believed to be synergistic regulation *via* both auxin and GAs (Fenn and Giovannoni, 2021). Fruit ripening requires a sequence of biochemical and physiological transformations. The highly dynamic development trajectories are tightly dependent on phytohormones (ABA and ethylene) crosstalk with epigenetic regulated gene expression (Giovannoni, 2004; Teyssier et al., 2015). Transcription factors started to emerge as recruitment of histone acetylation and deacetylation proteins by direct or indirect interaction to target gene promoters lead to changes in acetylation level, resulting alter gene expression (Servet et al., 2010).

During the fruit ripening process, based on the change of physiologies and in the action of ethylene, it is generally classified into climacteric fruit or non-climacteric fruit (Brumos, 2021). Ethylene plays a clear role in climacteric fruits during fruit ripening, whereas non-climacteric ripening is generally associated with ABA (Fenn and Giovannoni, 2021). Silencing of the HADC gene, *SlHDT3*, delays fruit ripening and suppresses carotenoid accumulation in tomato (Guo et al., 2017b). In tomato and apple, HDACs to deacetylation of ripening-related gene promoters, including ethylene biosynthetic genes to repress the ripening process (Deng et al., 2022; Hu et al., 2022), suggesting acetylation levels are associated with the onset of the climacteric fruit ripening process. However, whether HATs and HDACs are involved in regulating the non-climacteric fruit ripening process is unclear.

Pepper (*Capsicum* sp.) is a typical non-climacteric fruit. Due to its desired pigments, flavor, and aroma, pepper is cultivated and distributed worldwide (Zhu et al., 2019; Villa-Rivera and Ochoa-Alejo, 2021; Sun et al., 2022), about 54.9 million tons of green pepper and 4.1 million tons of dry pepper (FAO). The development and ripening of pepper fruits mainly include three steps (i.e., fruit set, fruit development, and fruit ripening) which are believed to be tightly associated with auxins, GAs, and ABA (Villa-Rivera and Ochoa-Alejo, 2021). Besides alters in fruit shape, changes in metabolism that occur during the ripening process of peppers cause transformations in flavor, color, texture, and aroma (Jang et al., 2015; Liu et al., 2019). A characteristic quality of pepper fruits is pungency, and they produce and accumulate hot compounds called capsaicinoids, which are alkaloids found exclusively in *Capsicum* species (Sun et al., 2022). *Capsicum* fruit capsaicinoids are genetically determined and depend on the developmental stage, and it occurs in the fruits from the early stages to mature green (Zhu et al., 2019; Arce-Rodríguez et al., 2021; Sun et al., 2022). Pepper ripening is accompanied by an accumulation of carotenoids. The different colors of ripening pepper fruits are due to variations in carotenoid composition and content in the pericarp. The capsanthin is the major red pigment of the fruits and accounts for 80% of the carotenoids in the high red intensity pepper cultivars (Berry et al., 2019). The capsanthin biosynthesis process is strictly switched spatially and temporally, and the carotenoid biosynthetic genes mainly transcript in fruit from the break stage to the mature stage (Kim et al., 2014; Song et al., 2020). Through pepper fruit development and ripening process highly dynamic regulated at the transcription level, whether HATs and HDACs are associated with fruit development and ripening remains unknown.

Compared to Arabidopsis and climacteric fruit tomato, relatively few HATs and HDACs were characterized in other plant species. In contrast to the start to the emergence of HATs and HDACs in controlling climacteric fruit development and ripening (Guo et al., 2017b; Deng et al., 2022; Hu et al., 2022), rare studies reported on the process of these functions in non-climacteric fruits. To obtain more insight into HATs and HDACs associated with non-climacteric fruit development and ripening. In this study, the HAT and HDAC families were identified in the non-climacteric fruit pepper genomes. The phylogenetic relationships, gene structure organization, chromosome distribution, conserved domain analysis, and motif protein composition were studied. Furthermore, the expression analysis during pepper fruit development identified *HAT* and *HDAC* genes that may play important roles in fruit development and ripening. This work provided insights into the function of *HAT* and *HDAC* in non-climacteric fruit development and ripening processes, and some of them may hold promise for fruit trait improvement in peppers.

## Materials and methods

### CaHATs and CaHDACs sequence analyses and characteristics

*CaHATs* and *CaHDACs* were retrieved from the ‘‘Zunla-1’’ (*C. annuum*) genome, which is available in the Solanaceae Genomics Network,^1^ by using the well-characterized HATs and HDACs from Arabidopsis and tomato as queries to search against the Solanaceae Genomics Network and NCBI database by the BLASTP program. The full-length amino acid sequences (length), theoretical isoelectric point (PI), and molecular weight (MW) instability index of ERF proteins were predicted by using the ExPASy server.^2^

### Sequence alignment and phylogenetic analysis

For the sequence and phylogenetic analysis, the protein sequences of HATs and HDACs from four species (i.e., *Capsicum annuum*, *Solanum lycopersicum*, *Arabidopsis thaliana*, and *Oryza sativa*) were adopted for analysis. The CaHAT and CaHDAC amino acid sequences were aligned using MUSCLE with the default parameters, respectively. Maximum likelihood trees were constructed with 5,000 bootstrap replications using IQ-tree after trimming the results of multiple sequence comparisons using trimAL (V1.2).

### Conserved motif and gene structure analysis

The conserved motif of CaHATs and CaHDACs was received by submitting protein sequences to the MEME Network^3^ with the following parameters: The maximum number of motifs for CaHATs is 30, and CaHDACs is 20. The use of NCBI tools is to discover the determination of conserved protein domains. Then, the results are visualized through TBtools (Chen C. et al., 2020).

### Genomic organization of CaHATs and CaHDACs

To determine the physical location of *CaHATs* and *CaHDACs*, the TBtools (Chen C. et al., 2020) was applied to locate the *CaHATs* and *CaHDACs* on “Zunla-1” pepper chromosomes according to their positions given in the genome database (Qin et al., 2014).

### Expression analysis of CaHATs and CaHDACs

The pepper fruit nine development stages of “Zunla-1” (Qin et al., 2014) and 11 development stages of “6,421” (Liu et al., 2017) RNA-seq data were retrieved from the database. Heat maps showing the expression patterns of genes were constructed with the R Programming Language. The Pearson correlation coefficient was applied to determine the gene expression relationship.

### Plant material

The “59” pepper inbred line with a high yield is resistant to a variety of pathogens and has strong resistance to abiotic stress. The seeds were sowed at the nursery site, and 35-day-old seedlings were transplanted into 35-cm non-woven pots. The plants were grown in a greenhouse with a daily temperature of 25–27°C, a nighttime temperature of 20–22°C, relative humidity of 60%, 16/8-h light/dark cycle, and light intensity of 6,500 Lux. During the pepper fruit was at the designed development stages, the fruits were sampled. The samples were frozen in liquid nitrogen and stored in a −80°C freezer.

### RNA extraction and RT-qPCR analysis

To verify the transcription level of *CaHATs* and *CaHDACs*, the RT-qPCR analysis was performed. Total RNA was extracted from the 6, 14, 25, 33, 38, and 48 DPA pepper fruit samples by using the HiPure HP Plant RNA Mini Kit (Magen, China). A total of 500 ng RNA were reverse-transcribed with the HiScript III 1st Strand cDNA Synthesis Kit (+ gDNA wiper) (Vazyme Biotech, China).

Quantitative real-time RT-PCR analysis was performed on a CFX384 Touch detection system (Bio-Rad, United States). The reaction mix was 0.5 μL of cDNA template, 0.2 μL of each primer (10 μmol/μL), 5 μL of SYBR Green Master Mix (Vazyme Biotech, China), and added nuclease-free water to 10 μL. The PCR amplification conditions were as follows: 95°C for 5 min; then 40 cycles at 95°C for 5 s; and 60°C for 30 s. The *ubiquitin extension gene* (*CA12g20490*) served as the reference gene for expression analysis (Liu et al., 2021). The relative expression is expressed as the target gene to the *ubiquitin extension gene* (*CA12g20490*). Each value represents the mean of three biological replicates. The *CaHATs* and *CaHDACs* RT-qPCR primer, which enables amplified target genes PCR product length, ranged from 100 to 300 bp. The primers used in this study are listed in Supplementary Table 2.

## Results

### Identification of CaHATs and CaHDACs in pepper genome

We used the protein sequences of 12 AtHATs from Arabidopsis (*Arabidopsis thaliana*) and 32 SlHATs from tomato (*Solanum lycopersicum*); 18 AtHDACs from Arabidopsis, and 14 SlHDACs from tomato as queries to search against the Solanaceae Genomics Network and NCBI database by the BLASTP program. As a consequence, 30 CaHATs and 15 CaHDACs were identified from the “Zunla-1” pepper genome after searching for HAT and HDAC domain sequences, respectively. The HATs and HDACs were named according to their subfamily on the position of the chromosome (Supplementary Table 1). The HAT protein length ranged from 157 aa (CaHAG18) to 1,856 aa (CaHAF1), pI ranged from 4.19 (CaHAG23) to 9.56 (CaHAG11), and MW ranged from 18.15 kDa (CaHAG18) to 210.08 kDa (CaHDA3). Concerning HDAC proteins, the length ranged from 217 aa (CaHDA1) to 675 aa (CaHDA3), pI ranged from 4.57 (CaHD3) to 8.98 (CaSRT2), and MW ranged from 24.01 kDa (CaHDA1) to 75.98 kDa (CaHDA3). In contrast to the conserved number of *HDAC*s in pepper (*Capsicum annuum*), Arabidopsis (*Arabidopsis thaliana*), tomato (*Solanum lycopersicum*), and rice (*Oryza sativa*), the *HATs* in these species seem subject to unequal duplication and evolution in which the number ranges from 8 (*Oryza sativa*) to 32 (*Solanum lycopersicum*).

### Phylogenetic of CsHATs and CsHDACs in pepper

To further investigate the evolutionary relationships and classification of CaHAT and CaHDAC members, the protein sequences from the pepper (*Capsicum annuum*), Arabidopsis (*Arabidopsis thaliana*), tomato (*Solanum lycopersicum*), and rice (*Oryza sativa*) were used for phylogenetic analysis. The result indicates the HATs from the four species were divided into seven clades.

The phylogenetic tree showed that 15 HDACs protein members in pepper were clearly classified into three subfamilies, 10 members belong to HDA1/RPD3 (Reduced Potassium Dependence 3/Histone Deacetylase 1) subfamily, 3 proteins belong to the plant-specific HD2 (Histone Deacetylase 2) subfamily, and 2 members belong to SIR2 (Silent Information Regulator 2) subfamily. The phylogenetic results consistent with HDAC were divided into three subfamilies, RPD3/HDA1, SIR2, and plant-specific HD2 (Guo et al., 2017a; Yuan et al., 2020).

### CsHATs and CsHDACs conserved domain

**FIGURE 1:**
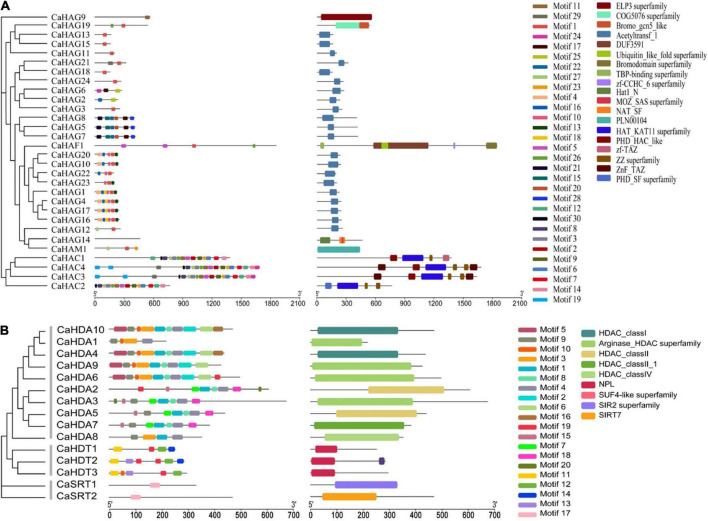
Phylogenetic tree of CaHAT **(A)** and CaHDAC **(B)** proteins associated with the motif compositions and conserved domain. The maximum likelihood method was applied for the construction phylogenetic tree. The number at each node represents the bootstrap value from 5,000 replicates. The motif composition and conserved domain of each CaHAT and CaHDAC protein are exhibited in the middle of the figure. Motifs and domains are displayed on the right of the figure. The length of the protein can be estimated from the scale provided at the bottom.

We identified the CaHAT and CaHDAC proteins through an online server (MEME). In total, 27 motifs were identified in 30 CaHATs and exhibited a high variation manner (Figure 1A). For the CaHATs, harboring motifs range from 1 to 18, whereas CaHAG14 did not present any motif. For these CaHATs, 19 conserved domains were identified, and CaHAT process conserved domains range from 1 to 6. Notably, most of the CaHATs presented the Acetyltransf_1 domain. The motifs of the 15 CaHDAC proteins displayed considerable variation, and 17 motifs were identified among these CaHDACs (Figure 1B). The motifs of the 15 CaHDAC proteins displayed considerable variation, in which motif number of CaHDAC proteins ranged from 1 to 10. Motif 4 was found in all CaHDA subgroup proteins. CaHDT subgroup was fond presented motifs 11, 12, and 19. CaSTR subgroup proteins only existed with one motif 17. Conserved domain analysis found HDAC domain exhibited in RPD3/HDA1 subgroup proteins, NPL domain presented in plant-specific CaHD2 subgroup proteins, and SIR2 and SIRT7 supper family domain only existed in SIR2 subgroup proteins. This result is highly consistent with the phylogenetic analysis.

### Chromosomal location of CaHATs and CaHDACs

**FIGURE 2:**
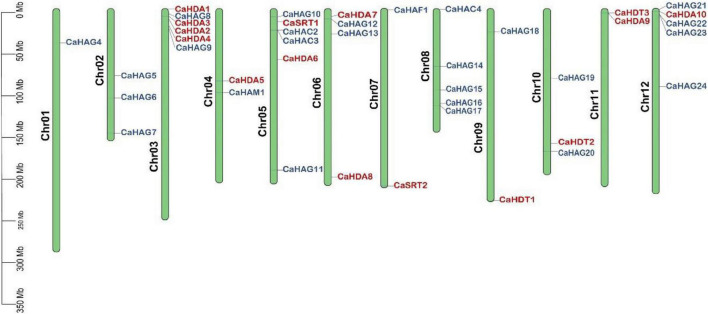
Chromosomal location of *CaHATs* and *CaHDACs* gene family. Four genes (*CaHAC1*, *CaHAG1*, *CaHAG2*, and *CaHAG3*) that did not assemble on the chromosome were omitted for display.

The identified pepper HATs and HDACs were mapped to chromosomes. However, four genes, including *CaHAG1*, *CaHAG2*, *CaHAG3*, and *CaHAC1*, were not anchored because the physical map of pepper was incomplete. Both *CaHATs* and *CaHDACs* were unevenly distributed on the chromosomes (Figure 2). There were no *CaHAT* genes on chromosomes 11, and chromosomes 1, 2, and 6 did not find *CaHDAC* genes. As to *CaHAG21*, *CaHAG22*, and *CaHAG23* were closely located on chromosome 12, while *CaHDA1*, *CaHDA2*, *CaHDA3*, and *CaHDA4* were closely located on chromosome 3, suggesting the occurrence of tandem duplication.

### Expression pattern of the CaHAT and CaHDAC gene family validates histone acetylation and deacetylation in fruit development

It has been reported that HAT and HDAC play key roles in plant growth and development (Pandey et al., 2002; Hu et al., 2009). To understand the expression profiles of *CaHAT* and *CaHDAC* genes in the pepper fruit developmental stage, the transcriptomic data of “Zunla-1” and “6,424” fruit’s different developmental stages were retrieved from the public database (Qin et al., 2014; Liu et al., 2017). The results showed that *CaHAT* and *CaHDAC* gene family expression patterns exhibited considerable variations. According to the hierarchical clustering results, the expression pattern of two gene families was mainly classified into the fruit early development stage upregulation group and the fruit late development upregulation stage group. For the early stage upregulation group, the *CaHATs* (*CaHAM1*, *CaHAG7*, *CaHA14*, *CaHAG5*, and *CaHAC4*) and *CaHDACs* (*CaHAD7*, *CaHAD10*, and *CaSTR2*) might play a crucial role in fruit growth, especially by regulate and/or interaction with phytohormone biosynthetic, receptor, signal response components to govern fruit cell division and expansion. In addition, some gene expression profiles showed more complicated, and the expression levels were considerable in fruit growth and ripening stages (*CaHAG11*, *CaHAG24*, *CaHAG13*; CaHDA2, and CaHDA8), suggesting these proteins process multiple functions in fruit. Regarding the late-stage upregulation group, *CaHATs* (*CaHAG3*, *CaHAG6*, *CaHAG9*, *CaHAG12*, *CaHAG15*, *CaHAG21*, *CaHAF1*, and *CaHAC1*) and *CaHDACs* (*CaHDA7*, *CaHAD9*, and *CaSTR2*) expression tightly associate with the fruit ripening process. To further reveal whether *CaHAT* and *CaHDAC* expression patterns among different cultivars, transcriptome data of the inbred line “6,421” fruit 11 developmental stages, which include pericarp, placenta, and seeds, respectively, were retrieved from previous studies (Liu et al., 2017). The results indicate though including different tissues, these *CaHATs* and *CaHDACs* are also mainly grouped into early development stage upregulation group and the late development stage upregulation group (Supplementary Figure 1). However, some *CaHATs* and *CaHDACs* are preferentially expressed in certain tissues, such as *CaHAG4* and *CaHDA4*, mainly expressed in the placenta. The tissue preferential expressed *CaHATs* and *CaHDACs* are likely to govern the tissue-specific biological process.

### The CaHAT and CaHDAC genes associated with fruit development

**FIGURE 3:**
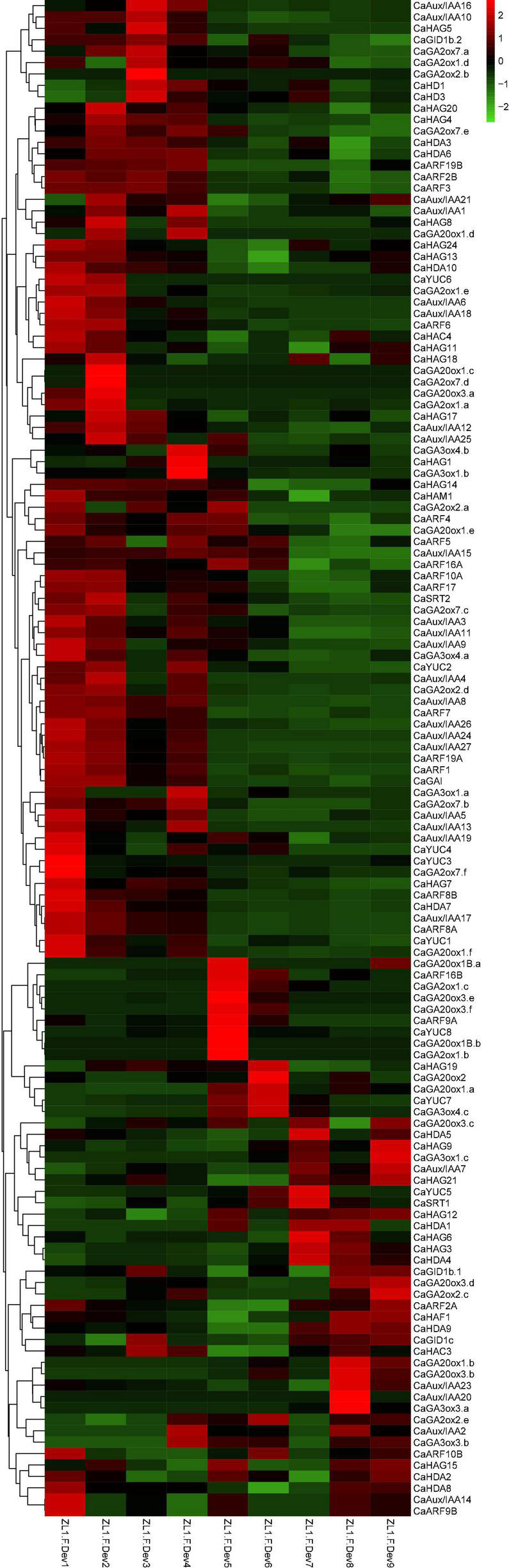
Hierarchical clustering analysis expression profiles of CaHATs, CaHDACs, auxin-, and GAs-related genes. The auxin-related genes include biosynthetic genes (*CaYUCs*) and signal transduction genes (*CaARFs* and *CaIAA/ARFs*). The GA-related genes include biosynthetic genes (subfamily of *CaGA2ox*, *CaGA3ox*, and *CaGA20ox*) and signal transduction genes (*CaGAIs* and *CaGIDs*). The heat map was constructed by values of fragments per kilobase of exon per million fragments (FPKM). The name of each gene is shown at the right of the heat map. The expression data were retrieved from “Zunla-1” transcriptome (Qin et al., 2014).

**FIGURE 4:**
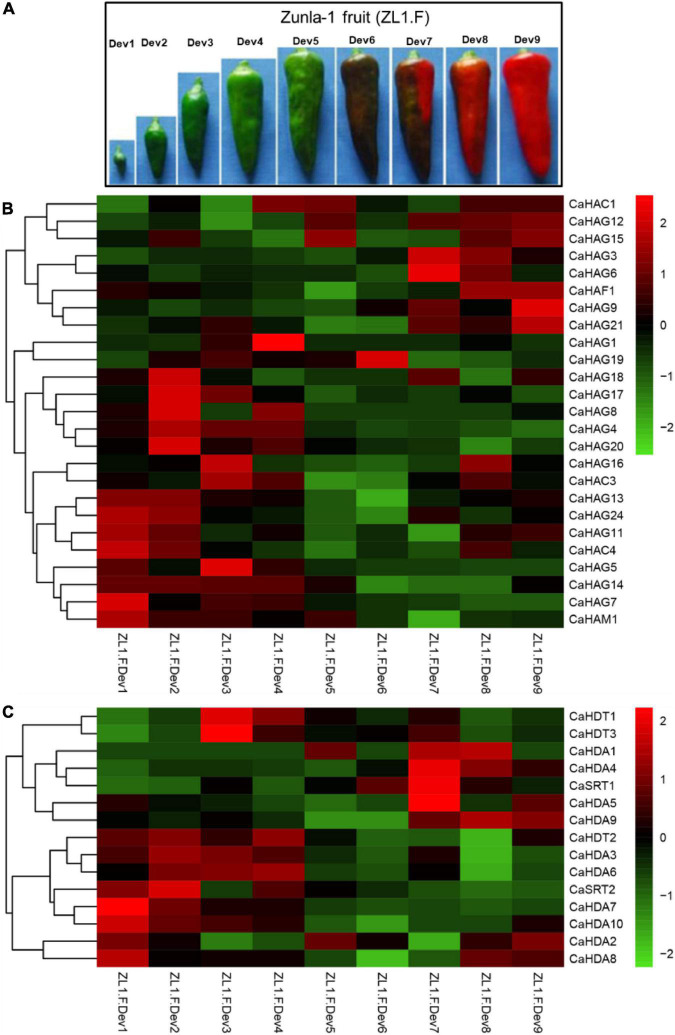
Expression pattern of CaHATs and CaHDACs in pepper nine fruit developmental stages. **(A)** “Zunla-1” (ZL-1) fruit at nine different stages, including the whole development stages of pepper fruit. The expression of *CaHATs* **(B)** and *CaHDACs* **(C)** at nine different development stages is shown in **(A)**. The expression data were retrieved from “Zunla-1” transcriptome (Qin et al., 2014).

The auxin and gibberellic acid (GA) signaling play a crucial role in fruit development (Fenn and Giovannoni, 2021). Fruit development consists of cell division and expansion, cell division is shown to be influenced by auxin signaling, and cell expansion is believed to be synergistic regulation *via* both auxin and GAs and auxin-responsive Aux/IAA, and ARF proteins modulate crosstalk of auxin and GAs. To clarify the potential relationship of *CaHAT* and *CaHDAC* between auxin and GAs biosynthetic, receptor, and signaling response genes, the expression and correlation of these genes were analyzed. The expression profile analysis indicates that most *CaAux/IAA* and *CaARF* are highly expressed in early development stage fruit (Figure 3), which exhibited similar profiles of early stage upregulation *CaHATs* and *CaHDACs* group (Figures 3, 4). In addition, Pearson’s correlation coefficient analysis indicates the early upregulation *CaHATs* (*CaHAG7* and *CaHAM1*) and *CaHDACs* (*CaHDA3*, *CaHDA6*, *CaHDA7*, and *CaHD2*) transcription levels were significantly correlated with the fruit development-related *Aux/IAA*, *ARF*, and GA signal gene (*CaGAI* and *CaGID1b.2*) (Supplementary Figures 2–6). These results suggest that early upregulation *CaHATs* (*CaHAG7* and *CaHAM1*) and *CaHDACs* might modulate these auxin-related and GAs-related genes coordinate to regulate fruit development.

### CaHAT and CaHDAC are associated with pepper fruit capsaicinoid biosynthesis

**FIGURE 5:**
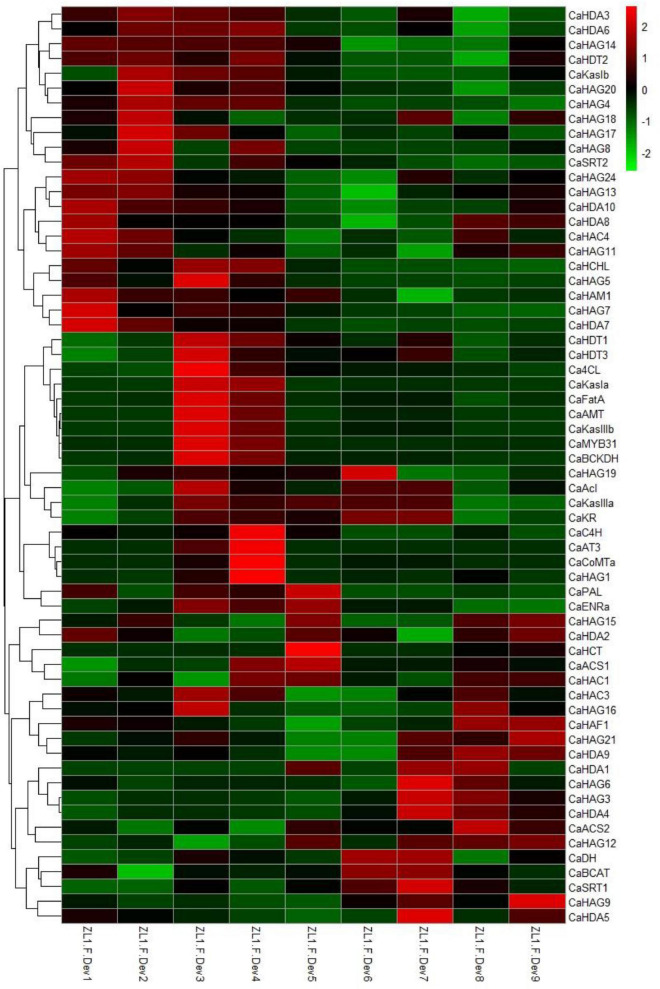
Expression profiles of *CaHATs*, *CaHDACs*, and capsaicinoid regulatory and biosynthetic genes. The heat map includes 19 capsaicinoid biosynthetic genes and one regulatory gene *CaMYB31*. The heat map was constructed by values of fragments per kilobase of exon per million fragments (FPKM). The expression data were retrieved from “Zunla-1” transcriptome (Qin et al., 2014).

The pepper characteristic metabolite capsaicinoid biosynthesis in the fruits from the early stage ZL.F.Dev3 reaches a maximum concentration at ZL.F.Dev6 (Qin et al., 2014). Consistent with capsaicinoid accumulation, the expression of the capsaicinoid biosynthetic genes is highly expressed from ZL.F.Dev3 to ZL.F.Dev4 (Figure 5). Moreover, the *CaHAG1* and *CaHAG19* of HATs and CaHDT1 and *CaHDT3* of HDACs also coexpressed with key capsaicinoid regulatory and biosynthetic genes (i.e., *CaMYB31*, *CaAT3*, *CaKasIa*, and *CaKasIIIb*) (Figures 5A,B). The results are also supported by Pearson’s correlation coefficient analysis (Supplementary Figures 7, 8). This result indicates these coexpressed *CaHATs* and CaHDACs are likely to regulate capsaicinoid biosynthetic gene histone acetylation levels to control gene expression.

### CaHATs and CaHDACs associated with pepper fruit ripening

**FIGURE 6:**
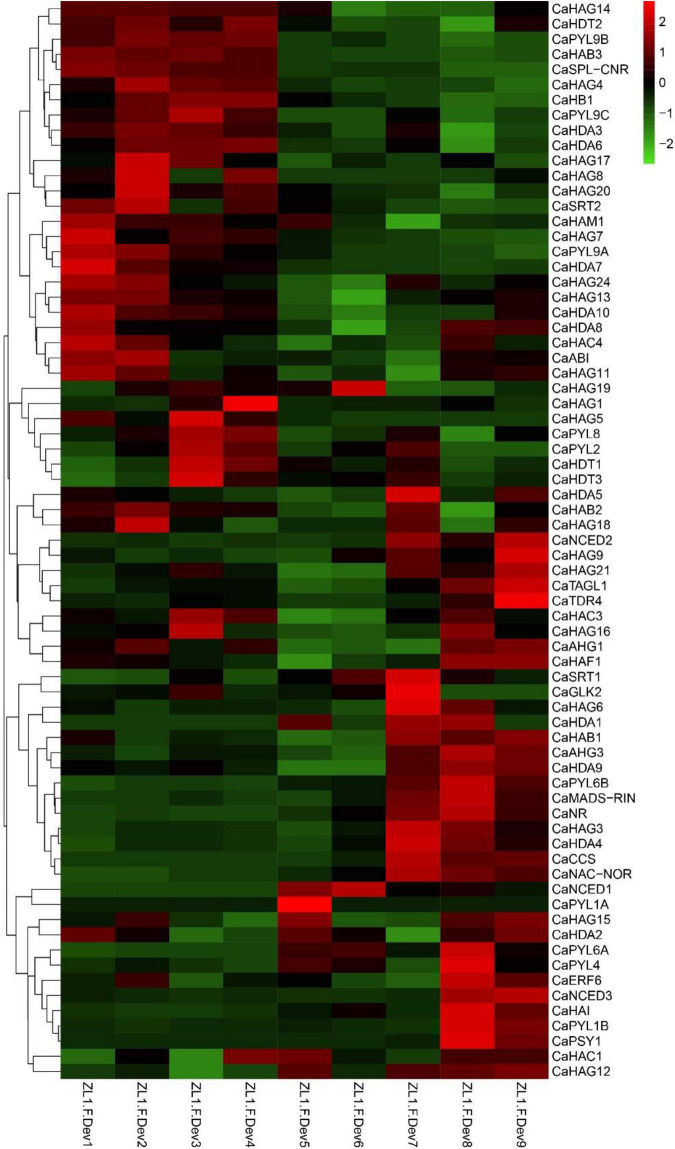
Expression profiles of *CaHATs*, *CaHDACs*, and ABA-related, ripening-related genes, and carotenoid biosynthetic genes. The ABA-related includes ABA biosynthetic (*CaNCEDs*) and signaling transduction genes (*CaPYLs*). Carotenoid biosynthetic genes include *CaPSY1* and *CaCCS*. The ripening-related genes were different transcription factors that govern the ripening process.

ABA is one of the main regulatory phytohormone in the ripening process of the non-climacteric fruits (Fenn and Giovannoni, 2021). Pepper is a typical non-climacteric fruit, in which fruit ripening is accompanied by the increased biosynthesis of the ABA (Xiao et al., 2020). To address the potential regulatory mechanism of *CaHATs* and *CaHDACs* in fruit ripening, the expression profiles of ABA biosynthesis, receptor, signal, and ripening-related genes, including carotenoid biosynthetic genes, were analyzed. The analysis of expression profiles uncovered that ABA-related (*CaNCED2/3, CaPLY4*, and *CaSnRK2.6*), ripening-related genes (*CaMADS-RIN*, *CaNR*, and *CaNAC-NOR*), and carotenoid biosynthetic genes (*CaPSY* and *CaCCS*) were mainly expressed in fruit’s late development stages (Figure 6). Notably, the late upregulation group *CaHATs* and *CaHDACs* such as *CaHAG3*, *CaHAG9*, and *CaHAG12* of *CaHATs* and *CaHDA1*, *CaHDA3*, *CaHDA5*, and *CaHDA9* of *CaHDACs* are tightly coexpressed with these ABA-related, ripening-related genes and carotenoid biosynthetic genes (Figure 6), suggesting these histone acetylation modifiers mediate certain of these genes acetylation level to control fruit ripening.

### RT-qPCR analysis validates CaHATs and CaHDACs expression in pepper fruit in different development stages

**FIGURE 7:**
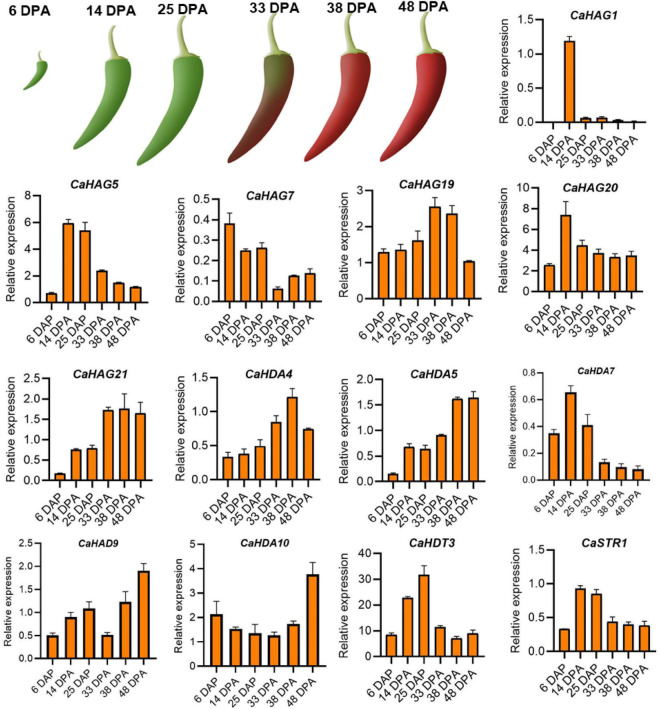
qRT-PCR analysis expression patterns of 13 *CaHAT* and *CaHDAC* genes in different developmental stages of the fruit. The upper figure indicates schematic diagram of “59” inbred line fruit phenotypes at six developmental stages: 6, 14, 25, 33, 38, and 48 DPA. Bottom of figure indicates the expression of 13 selected *CaHATs* and *CaHDACs* expression patterns in fruit six developmental stages. Data are mean ± SE (*n* = 3).

For validating the expression patterns of the histone acetylation modifiers consistent with pepper fruit development and ripening, in total, 13 of *CaHATs* (*CaHAG1*, *CaHAG5*, *CaHAG7*, *CaHAG9*, *CaHAG20*, and *CaHAG21*) and *CaHDACs* (*CaHDA4*, *CaHDA5*, *CaHDA7*, *CaHDA9*, *CaHDA10*, *CaHDT3*, and *CaSTR1*) were adopted for analysis, respectively. The total RNA was extracted from “59” inbred line fruit at the development stage of 6, 14, 25, 33, 38, and 48 DPA (corresponding to the “Zunla-1” development stage of ZL-1.F.Dev1, ZL-1.F.Dev3, ZL-1.F.Dev4, ZL-1.F.Dev6, ZL-1.F.Dev7, and ZL-1.F.Dev9). The RT-qPCR analysis indicates that these genes’ relative expression levels were development-dependent (Figure 7). We found some *CaHATs* (*CaHAG1*, *CaHAG5*, *CaHAG7*, *CaHAG9*, and *CaHAG20*) and *CaHDACs* (*CaHDA7*, *CaHDT3*, and *CaSTR1*) mainly expressed in the fruit early stage, suggesting these are involved in fruit development. However, other *CaHATs* (*CaHAG19* and *CaHAG21*) and *CaHDACs* (*CaHDA4, CaHDA5, CaHDA9*, and *CaHDA10*) were mainly expressed in the fruit later stages, implying they are associated with the ripening process. The RT-qPCR analysis was consistent with the transcriptome results of pepper cultivar “Zunla-1” and “6,421” fruits, suggesting the gene expression was genotype-independent and strongly supported their association with fruit development and ripening.

## Discussion

*HAT* and *HDAC* gene families have been identified in different species, including Arabidopsis (Pandey et al., 2002), rice (Hu et al., 2009), grapevine (Aquea et al., 2010), tomato (Aiese Cigliano et al., 2013), Citrus (Xu et al., 2015; Shu et al., 2021), litchi (Peng et al., 2017), tea plant (Yuan et al., 2020), and winter wheat (Li et al., 2021, 2022). In contrast relative conserved number of *HDAC* in different species, whereas *HAT* exhibited considerable variation. In this study, we identified 32 *CaHATs* and 15 *CaHDACs* in the pepper genome (Table 1). Notably, compared with other species, the *HATs* subfamily *HAGs* have remarkably expanded in pepper (24 *HAGs*), and tomato (26 *HAGs*) compared with dicotyledonous Arabidopsis (3 *HAGs*) and monocotyledonous rice (3 *HAGs*) (Table 1). In plants, the gene family expansion mainly arises from tandem duplications and segmental or whole-genome duplications (Freeling, 2009). Presumably, after being separated from their ancestors, *HATs* likely undergo unequal duplication events among species. Indeed, location analysis found some Ca*HATs* (such as *CaHAG21*, *CaHAG22*, and *CaHAG23*) were closely located on chromosomes, suggesting the expansion of these genes arises from tandem duplications. Apparently, before tomato and pepper speciation, tandem duplications and segmental occurred in common ancestors, which finally led to *HATs* significantly expanding in Solanaceae.

**TABLE 1:** Number of HATs and HADCs in four plant species.

Types	Subfamily	Species			
		*Capsicum annuum*	*Solanum lycopersicum*	*Arabidopsis thaliana*	*Oryza sativa*
HATs	HAC	4	4	5	3
	HAG	24	26	3	3
	HAF	1	1	2	1
	HAM	1	1	2	1
Total		30	32	12	8
HDACs	HDA/RPD3	10	9	12	14
	HDT/HD2	3	3	4	2
	STR/SIR2	2	2	2	2
Total		15	15	18	18

**FIGURE 8:**
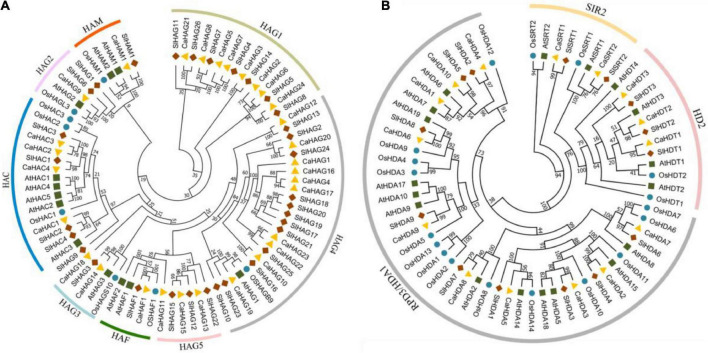
Phylogenetic tree of the HAT and HDAC proteins. The HAT **(A)** and HDAC **(B)** proteins of pepper (*Capsicum annuum*), Arabidopsis (*Arabidopsis thaliana*), tomato (*Solanum lycopersicum*), and rice (*Oryza sativa*) were adopted for analysis. The tree was constructed with the maximum likelihood method by adopting HAT and HDAC protein full-length protein sequences from pepper (*Capsicum annuum*), Arabidopsis (*Arabidopsis thaliana*), tomato (*Solanum lycopersicum*), and rice (*Oryza sativa*). The number at each node represents the bootstrap value from 1,000 replicates. The protein sequences used in this study were retrieved from Solanaceae Genomics Network and NCBI database.

CaHATs and CaHDACs included subfamily-specific domains, and subfamily members also exhibited similar protein sequence length, motif composition, and gene structure, reflecting a close phylogenetic relationship. In this study, the HAT proteins could be divided into four families: HAC, HAF, HAM, and HAG (Figures 1, 8), consistent with previous studies (Pandey et al., 2002; Aquea et al., 2010; Aiese Cigliano et al., 2013; Li et al., 2021; Shu et al., 2021). The largest subfamily of CaHATs contained 24 CaHAGs and was further divided into five groups. After analyzing their protein structures, we found that the structures of HAGs were quite different, indicating that they might execute different functions and support the proposed subgroups. The winter wheat genome identified nine HAGs, and their protein structures are highly variable, enabling these HAGs into three groups (Li et al., 2022). In this study, the CaHDACs were classified into three subfamilies: HDA/RPD3, HDT/HD2, and STR/SIR2 (Figures 1, 8 and Table 1). The results are consistent with the identified HDACs in the genome of Arabidopsis (Pandey et al., 2002), rice (Hu et al., 2009), grapevine (Aquea et al., 2010), tomato (Aiese Cigliano et al., 2013), Citrus (Xu et al., 2015), papaya (Fu et al., 2019), and tea plant (Yuan et al., 2020), but the HDT/HD2 subfamily orthologous seems to have been lost in the litchi genome during the duplication events (Peng et al., 2017). Whether these HDACs process similar functions among different species needs further study.

**FIGURE 9:**
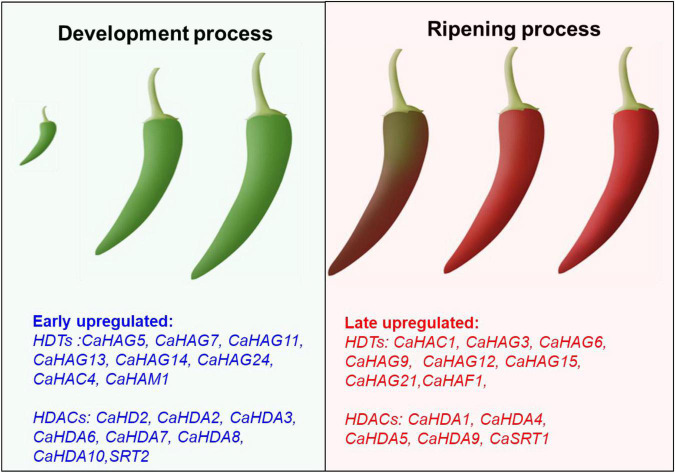
Proposal model of *CaHDTs* and *CaHDACs* candidates in pepper development and ripening. The leaf panel display early upregulated histone acetylation modifiers might be associated with the fruit development-related process. The right panel display late upregulated histone acetylation modifiers might be related to the fruit ripening-related process.

Histone modifications play a critical role in growth, development processes, flowering, and stress responses (Pandey et al., 2002; Hu et al., 2009). Recently, based on stress treatment-derived gene expression profiles, in tea plant (Yuan et al., 2020), *Citrus sinensis* (Shu et al., 2021), and winter wheat (Li et al., 2022), we identified a set of stress-inducible *HATs* and *HDACs* and proposed these genes play an essential role in stress response. In this study, we identified 32 *CaHATs* and 15 *CaHDACs*, but the specific functions of HATs and HDACs in pepper remain unknown. Based on the expression profiles, the *CaHATs* and *CaHDACs* were mainly classified into the fruit early development stage upregulation group and the late development stage upregulation group (Figures 4, 9). The fruit’s early un-regulation group histone modifiers were proposed to regulate fruit development (including cell division and expansion) for their highly coexpressed with auxin and GAs biosynthetic and signal response genes (Figure 3). In addition to changes in fruit size, pepper development also along with capsaicinoid biosynthesis. Notably, some *CaHATs* and *CaHDACs* display similar profiles with capsaicinoid biosynthetic genes (Figure 5). Perhaps, the mechanism of CaHATs and CaHDACs controls fruit development and capsaicinoid biosynthesis on the two sides. On the one hand, histone modifiers alter acetylation level to regulate development-related genes (including auxin and GAs pathway-related genes) and capsaicinoid biosynthetic gene expression by changing the state of the chromatin; on the other hand, CaHATs and CaHDACs recruiting DNA-binding transcription factor to formate protein complexes affect target genes expression. It can be learned from Arabidopsis that a light-regulated histone deacetylase HDA15 interacts with PIF1to suppress the light-responsive genes during seed germination in the dark (Gu et al., 2017) and HDA15 also restricts chlorophyll biosynthesis- and photosynthesis-related genes through its interaction with PIF3 (Liu et al., 2013). Likewise, HDA19 interacts with histone methyltransferase SUVH5 and regulates seed dormancy through ABA and GA signaling pathways by modulating overall histone H3 acetylation and H3K9me2 methylation on the promoter of the target genes *ABI3*, *RGA*, and *DOG1* (Zhou et al., 2020; Kumar et al., 2021). The Arabidopsis MYST histone acetyltransferase HAM1/2 enhances the expression of the negative regulator FLC gene through H4K5 acetylation and results in delayed flowering (Xiao et al., 2013). The cucumber (*Cucumis sativus*) *SF2* encodes a Histone Deacetylase Complex1 (HDC1) homolog and its role in regulating cell proliferation through the HDAC complex to promote histone deacetylation of key genes involved in multiple phytohormone pathways and cell cycle regulation (Zhang et al., 2020). A weak *sf2* allele impairs HDAC targeting to chromatin, resulting in elevated histone acetylation levels. Overall, these studies indicate histone acetylation plays a critical role in development, whereas the exact regulatory mechanism concerning CaHATs and CaHDACs control fruit development and capsaicinoid biosynthesis needs further investigation. In addition, CaHATs and CaHDACs cannot bind DNA directly, identifying the key transcription factors involved in plant development may interact, and recruiting these histone modifiers in the co-regulation of gene expression is needed.

Compared to the rare study focused on fleshy fruit development, numerous studies have reported histone acetylation involvement in the fruit ripening of tomato (Guo et al., 2017c,^2018^; Deng et al., 2022), bananas (Han et al., 2016; Fu et al., 2018), papaya (Fu et al., 2019), and pear (Li X. et al., 2020) and apple (Hu et al., 2022). However, most of the reported studies were relevant to histone acetylation involvement in climacteric fruit ripening, while this process control in non-climacteric fruit ripening remains elusive. In this study, we identified fruit late upregulation *CaHATs* and *CaHDACs* and regarded them as ripening candidates (Figures 6, 7, 9). Expression analysis found that they are highly coexpressed with ABA, ripening, and carotenoid-related genes, which supported their association with maturation. The orthologous HAF in tomato (*SlHAF1*) (Xu et al., 2015) and Citrus (*CsHAF1/2*) has the strongest expression in mature fruit, suggesting an important role in ripening. Similarly, the identified *CaHAF1* also showed a high expression level in pepper breaking and ripening stage fruit (Figure 4), implying that they could have similar functions as tomato and Citrus. Tomato SlHDT1 and SlHDT3 positively regulate ripening by regulating carotenoid and ethylene content and expression of ethylene biosynthetic genes, ripening-associated genes, and cell wall metabolism genes (Guo et al., 2017b; Guo, 2022). The *CaHDT1* and *CaHDT3* were highly expressed in pepper immature and mature fruit (Figure 4), suggesting it might control fruit ripening but also process other functions. In contrast, SlHDA1 and SlHDA3 process redundancy function and play a role in the repression of fruit ripening and carotenoid accumulation (Guo et al., 2017c,^2018^). While CaHDA3 is expressed in different development stages, HDA1 orthologous CaHDA1 is mainly expressed in pepper ripening fruit, implying the function suffers divergent evolution. Recently, studies found that HATs recruit coactivator proteins to activator gene transcription (Servet et al., 2010), whereas HDACs associated with corepressor proteins always form the complex to repress transcription (Kumar et al., 2021). Banana ripening negative regulator MaERF11 physically interacts with a histone deacetylase MaHDA1, and the interaction significantly strengthens the MaERF11-mediated transcriptional repression of MaACO1 and expansins (Han et al., 2016). Likewise, CpHDA3 is associated with CpERF9 in the suppression of CpPME1/2 and CpPG5 genes during papaya fruit ripening (Fu et al., 2019). Recently, the tripartite complex MdERF4–MdTPL4-MdHDA19 in apples (Hu et al., 2022) and SlERF.F12-SlTPL3-SlHDA1/3 in tomatoes (Deng et al., 2022) negatively regulate the onset of fruit ripening represses transcription of ripening genes by decreasing the level of the permissive histone acetylation marks H3K9Ac and H3K27Ac at their promoter regions. However, we should be kept in mind that all the mentioned above are climacteric fruit, and ethylene plays an essential role in the onset ripening process (Fenn and Giovannoni, 2021), whereas pepper is the non-climacteric fruit ripening, and ABA is responsible for ripening (Xiao et al., 2020). Whether orthologous climacteric fruit ripening-related *HDAC* genes process conserved functions in pepper needs further research. As another important modification, histone methylation, and demethylation have been evidenced to play an important role in fruit ripening by modulating H3K27 to regulate ripening-related gene expression (Li Z. et al., 2020; Ding et al., 2022); the exact function of histone methylation in pepper ripening also needs further study. In addition, fruit ripening along with genome-wide dynamic changes in DNA methylation has been identified in tomato (Zhong et al., 2013), strawberry (Cheng et al., 2018), and orange (Huang et al., 2019), and whether DNA methylation is associated with pepper fruit ripening should be dissected in future. Moreover, how histone modification and DNA methylation cooperate to regulate ripening-associated gene expression requires further investigation.

## Data availability statement

Publicly available datasets were analyzed in this study. This data can be found here: NCBI, SRP018258. The names of additional repositories and accession number(s) can be found in the article/Supplementary material.

## Author contributions

ZZ conceived and supervised the project, designed the study, and wrote the manuscript. GC provided some construction suggestions and revised the manuscript. YC, MX, JRL, and HZ performed major experiments and analyzed the data. GC, CC, BC, BS, QD, JJL, MC, and KC revised the manuscript, provided some useful suggestions, and revised the manuscript. All authors contributed to the article and approved the submitted version.

## Footnotes

1
https://solgenomics.net/

2
http://www.expasy.org/

3
https://meme-suite.org/meme/tools/meme
