# Supplementary material for: Genome-wide analysis of histone acetyltransferase and histone deacetylase families and their expression in fruit development and ripening stage of pepper (Capsicum annuum)
Source: Front Plant Sci. 2022 Sep 7;13:971230. doi: 10.3389/fpls.2022.971230 (PMC9490122; doi:10.3389/fpls.2022.971230)
Supplement: Supplementary file 1 [file Data_Sheet_1.docx]

**Genome-wide analysis of histone acetyltransferase (HAT) and histone deacetylase (HDAC) families and their expression in pepper (*Capsicum annuum*) fruit development and ripening stage**

Yutong Cai^1*^, Mengwei Xu ^1*^, Jiarong Liu^1*^, Haiyue Zeng^3, 4*^, Jiali Song, Binmei Sun^1^, Siqi Chen^1^, Qihui Deng^1^, Jianjun Lei^1^, Bihao Cao^1^, Changming Chen^1^, Guoju Chen^1^, Zhangsheng Zhu^1, 2, 4^

1. Key Laboratory of Biology and Germplasm Enhancement of Horticultural Crops in South China, Ministry of Agriculture and Rural Areas, College of Horticulture, South China Agricultural University, Guangzhou 510642, China

2. Guangdong Laboratory for Lingnan Modern Agriculture, Guangzhou 510642, China

3. School of Advanced Agricultural Sciences, Peking University, Beijing 100871, China

4. Peking University Institute of Advanced Agricultural Sciences, Weifang 261325, China

**^*^**These authors contributed equally to this study: Yutong Cai, Mengwei Xu, Jiarong Liu, Haiyue Zeng,

Correspondence Author: zhuzs@scau.edu.cn and gjchen@ scau.edu.cn

Supplementary Table 1 The details of pepper HATs and HADCs

| Subfamily | Gene name | Gene ID | | Protein length | MW | PI |
| --- | --- | --- | --- | --- | --- | --- |
| *HADCs* family  RPD3/HDA1 | CaHDA1  CaHDA2 | Capana03g000041  Capana03g000661 | | 217  608 | 24.01  66.54 | 5.51  5.22 |
|  | CaHDA3 | Capana03g000880 | | 675 | 75.98 | 5.64 |
|  | CaHDA4 | Capana03g001204 | | 438 | 48.97 | 5.47 |
|  | CaHDA5 | Capana04g001713 | | 441 | 47.79 | 6.62 |
|  | CaHDA6 | Capana05g001085 | | 498 | 55.77 | 5.23 |
|  | CaHDA7 | Capana06g000636 | | 383 | 41.80 | 5.25 |
|  | CaHDA8 | Capana06g002830 | | 353 | 39.21 | 6.86 |
|  | CaHDA9 | Capana11g000220 | | 426 | 48.43 | 5.07 |
|  | CaHDA10 | Capana12g000224 | | 471 | 52.99 | 5.31 |
| HD2 | CaHDT1 | Capana09g002339 | | 252 | 26.65 | 5.24 |
|  | CaHDT2 | Capana10g001577 | | 286 | 31.04 | 4.71 |
|  | CaHDT3 | Capana11g000213 | | 296 | 32.23 | 4.57 |
| SIR2 | CaSRT1 | Capana05g000570 | | 331 | 36.99 | 8.60 |
|  | CaSRT2 | Capana07g002373 | | 470 | 52.16 | 8.98 |
| *HATs* family |  | |  |  |  |  |
| GNAT | CaHAG1 | | Capana00g000321 | 228 | 26.44 | 6.32 |
|  | CaHAG2 | | Capana00g003272 | 235 | 27.12 | 8.33 |
|  | CaHAG3 | | Capana00g003720 | 255 | 28.14 | 6.99 |
|  | CaHAG4 | | Capana01g001284 | 246 | 28.10 | 5.5 |
|  | CaHAG5 | | Capana02g000664 | 414 | 46.83 | 9.35 |
|  | CaHAG6 | | Capana02g001118 | 276 | 31.97 | 9.38 |
|  | CaHAG7 | | Capana02g002984 | 417 | 46.61 | 8.81 |
|  | CaHAG8 | | Capana03g000470 | 407 | 45.99 | 7.52 |
|  | CaHAG9 | | Capana03g001394 | 565 | 63.47 | 8.87 |
|  | CaHAG10 | | Capana05g000449 | 242 | 28.06 | 5.9 |
|  | CaHAG11 | | Capana05g002125 | 200 | 22.05 | 9.56 |
|  | CaHAG12 | | Capana06g000810 | 261 | 29.98 | 8.96 |
|  | CaHAG13 | | Capana06g001413 | 166 | 18.68 | 8.96 |
|  | CaHAG14 | | Capana08g000464 | 464 | 53.19 | 6.15 |
|  | CaHAG15 | | Capana08g000666 | 161 | 18.38 | 8.9 |
|  | CaHAG16 | | Capana08g000827 | 250 | 28.30 | 5.99 |
|  | CaHAG17 | | Capana08g000842 | 245 | 28.02 | 6.17 |
|  | CaHAG18 | | Capana09g000635 | 157 | 18.15 | 9.22 |
|  | CaHAG19 | | Capana10g001000 | 543 | 60.92 | 5.77 |
|  | CaHAG20 | | Capana10g001723 | 238 | 26.36 | 5.96 |
|  | CaHAG21 | | Capana12g000117 | 318 | 37.18 | 9.26 |
|  | CaHAG22 | | Capana12g000365 | 192 | 22.20 | 5.3 |
|  | CaHAG23 | | Capana12g000368 | 201 | 23.04 | 4.19 |
|  | CaHAG24 | | Capana12g001429 | 269 | 29.69 | 9.41 |
| CBP | CaHAC1 | | Capana00g002880 | 1383 | 156.60 | 6.31 |
|  | CaHAC2 | | Capana05g000715 | 769 | 89.07 | 6.36 |
|  | CaHAC3 | | Capana05g000721 | 1648 | 185.94 | 8.52 |
|  | CaHAC4 | | Capana08g000069 | 1690 | 188.97 | 8.39 |
| MYST | CaHAM1 | | Capana07g000055 | 443 | 51.15 | 5.44 |
| TAFII250 | CaHAF1 | | Capana04g001771 | 1856 | 210.08 | 5.44 |

Supplementary Table 2 Primer sequence

|  | Forward | Reverse |
| --- | --- | --- |
| CaHDA7 | AAGGTCGCCCTGCTCAAGTCT | GACCGGCATTGTTAAGGAAGC |
| CaHD1 | CGTACACTTTACCCTCCAGACC | TATTCACGGACAAGCAGACATT |
| CaHDA10 | ACTTTCACAGTTACCTCATGCTCCTA | TCATCTCCATTGGCTTGGTTGT |
| CaHDT3 | CGAGGATAGACCTCAGTTGATG | CTCATCCTTAACAAGTTCAGACTTC |
| CaHDA5 | GTTTCGAGGTTTAGATGTTGTTG | GACTCTTGGAATGTTGTGGC |
| CaHDA4 | GCGATTATTACTACGGGCAAGG | AGGGCAATCCGAACCAACA |
| CaHDA9 | TTCCTGGTGGGCACATAGAG | TCGCCATCATAATACTCGTCATC |
| CaHAG5 | CTTAGTCCAACTGAAGCCGAAAT | TTGACGCACCACGAACCTC |
| CaHAG19 | CGATGATGACGAGGAAGAT | CAACATTACCGCAACAACC |
| CaHAG1 | AAACTAACCCTAAACCTCT | AATGCGTATCCAGCAATAT |
| CaHDT1 | TACACTTTACCCTCCAGACC | TTCACGGACAAGCAGACAT |
| CaSRT1 | TGCATGGGACTGTCTACGT | CAGAACTCCTTGGCACCTC |
| CaHAG7 | GCCATTTGGGCTTCACT | TCAACGGTTCCCTGCTT |
| CaHAG20 | TATTCACAAACTCATCCACCAA | TCGGGACATCAATCATTACG |
| CaHAG21 | TGAAGTACATTGCAAGAATGATC | CATGCCTCATATAGGACACAGAC |
| CaHAG3 | ATGCGGTTCCTGTCAAT | TCCCAGCGATACGTTCT |
| CaHAG9 | GAATGTGACTTGCCCAGAA | CGTCCCTACACCCGAAAT |


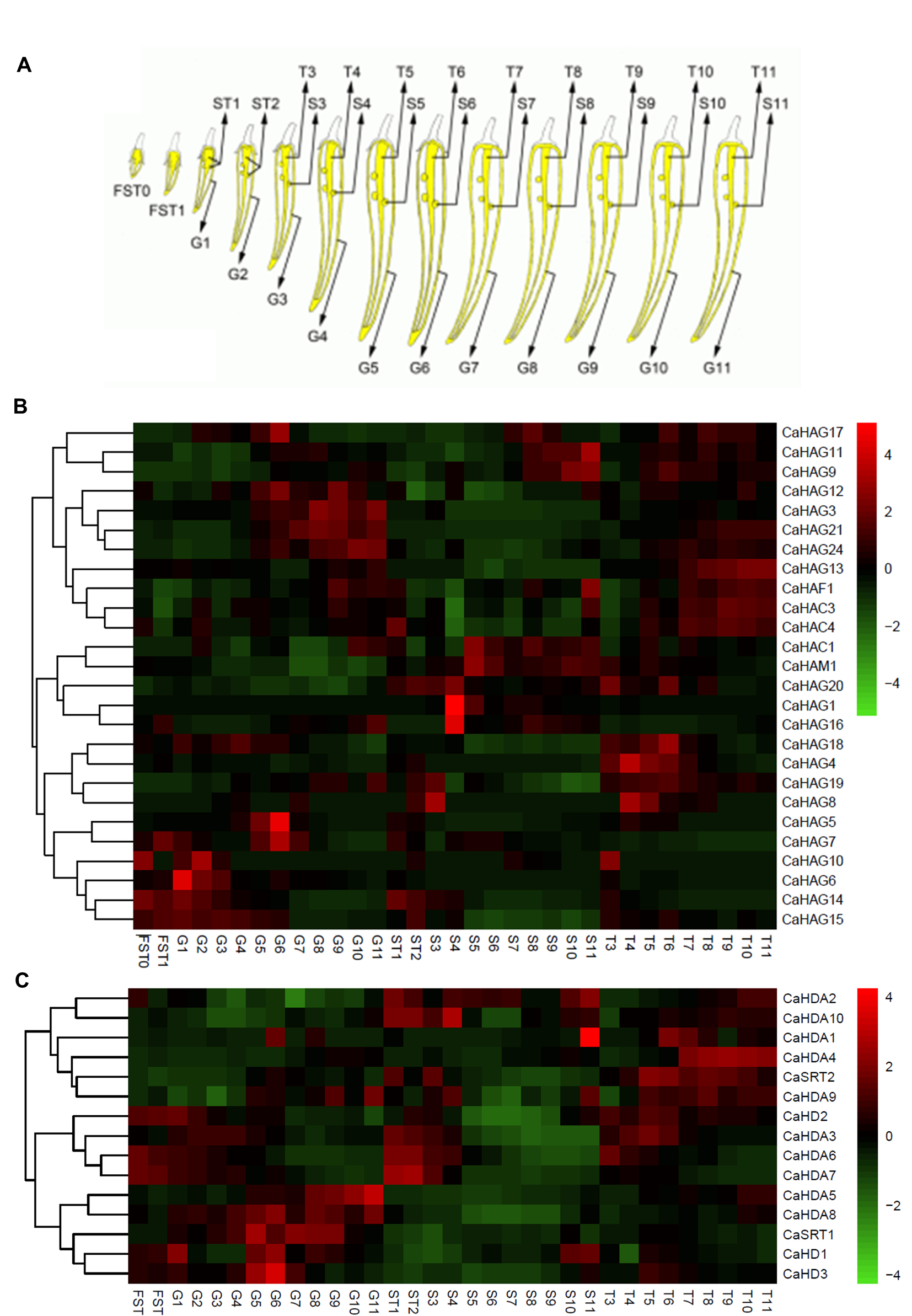


Supplementary Fig. 1 The *CaHAT* and *CaHDAC* genes family expression in line 6421 (*Capsicum annuum*) different development stages. (A) The line 6421 fruit development stages and sampling schematic diagram. (B) *CaHAT* genes family expression profiles in line 6421 fruit differental development stages. (C) *CaHDAC* genes family expression profiles in line 6421 fruit differental development stages.


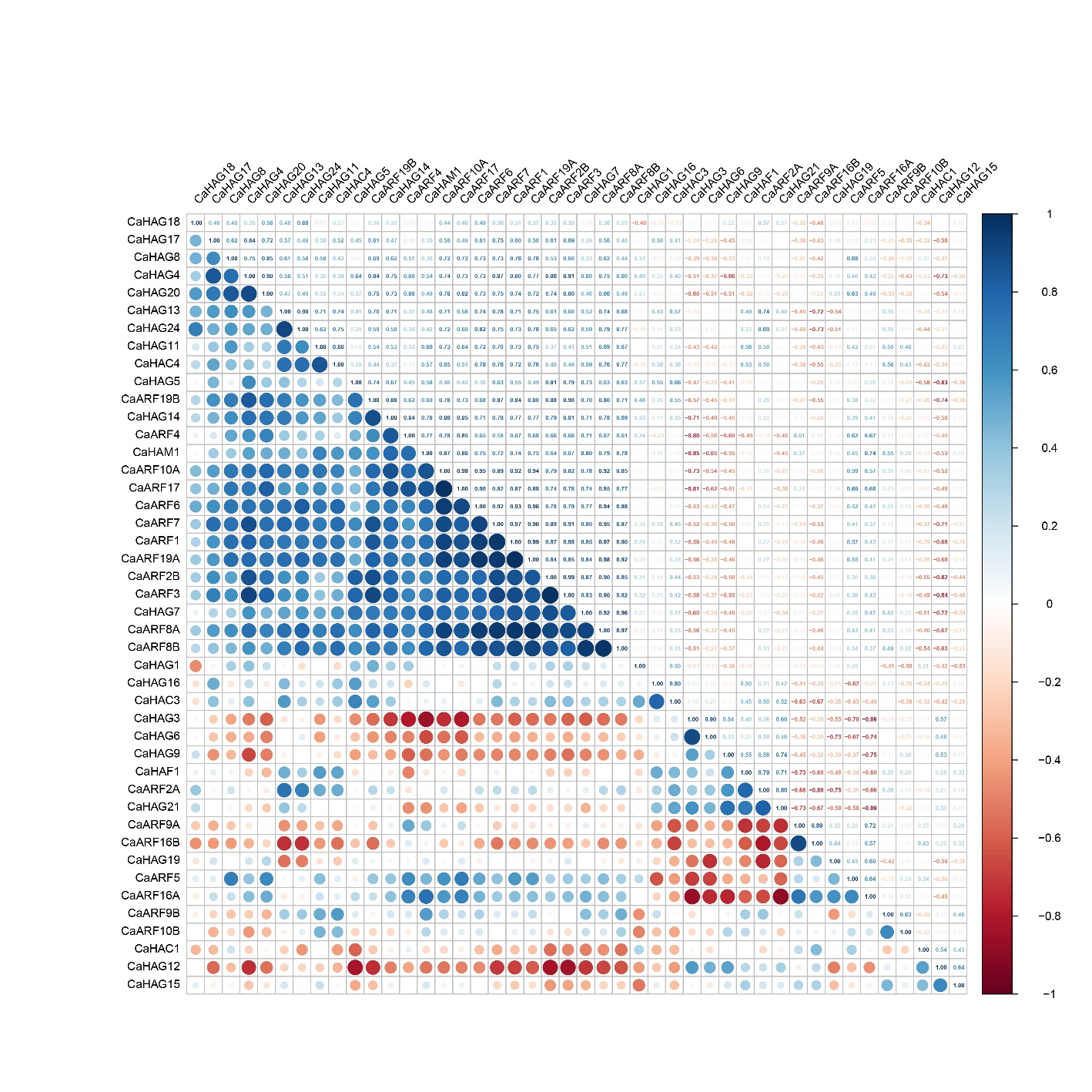


Supplementary Fig. 2 Pearson’s correlation coefficients for the *CaHAT* and *CaARF* genes’ transcription level. The expression data was retrieved from ‘Zunla-1’ transcriptome data of nine fruit developmental stages.


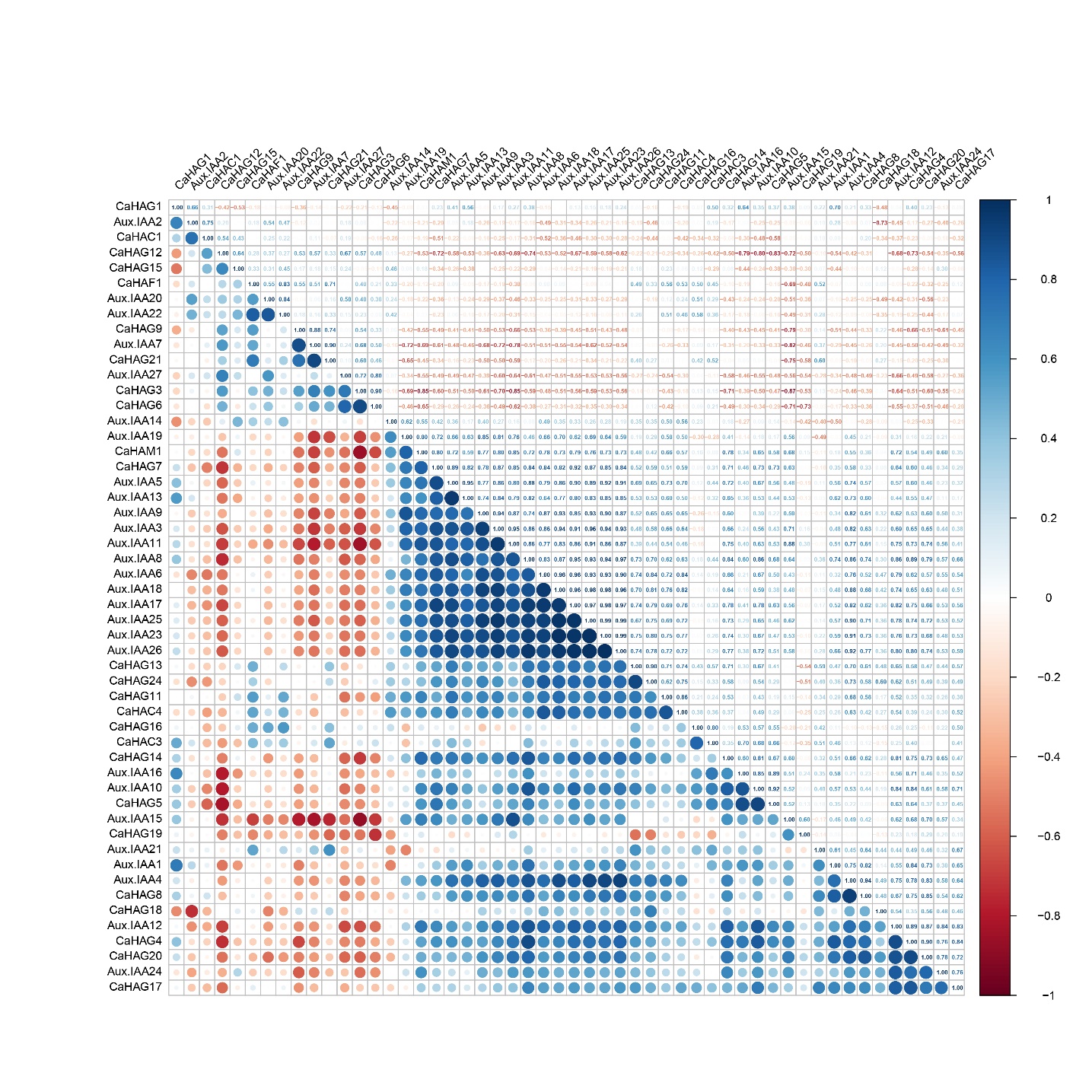


Supplementary Fig. 3 Pearson’s correlation coefficients for the CaHAT and Aux/IAA genes’ transcription level. The expression data was retieved from ‘Zunla-1’ transcriptome data of nine fruit developmental stages.


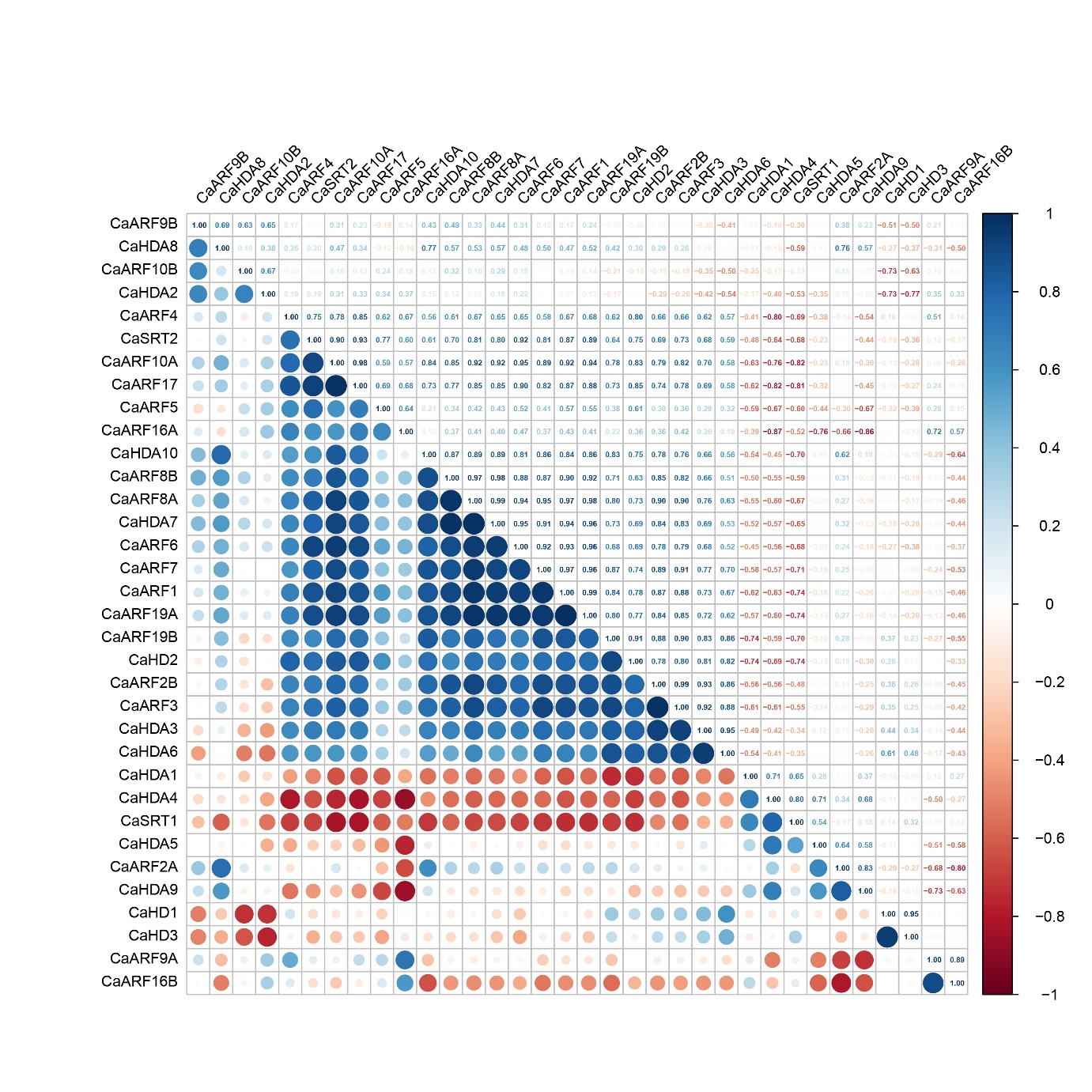


Supplementary Fig. 4 Pearson’s correlation coefficients for the *CaHDAC* and *CaARF* genes’ transcription level. The expression data were retrieved from ‘Zunla-1’ transcriptome data of nine fruit developmental stages.


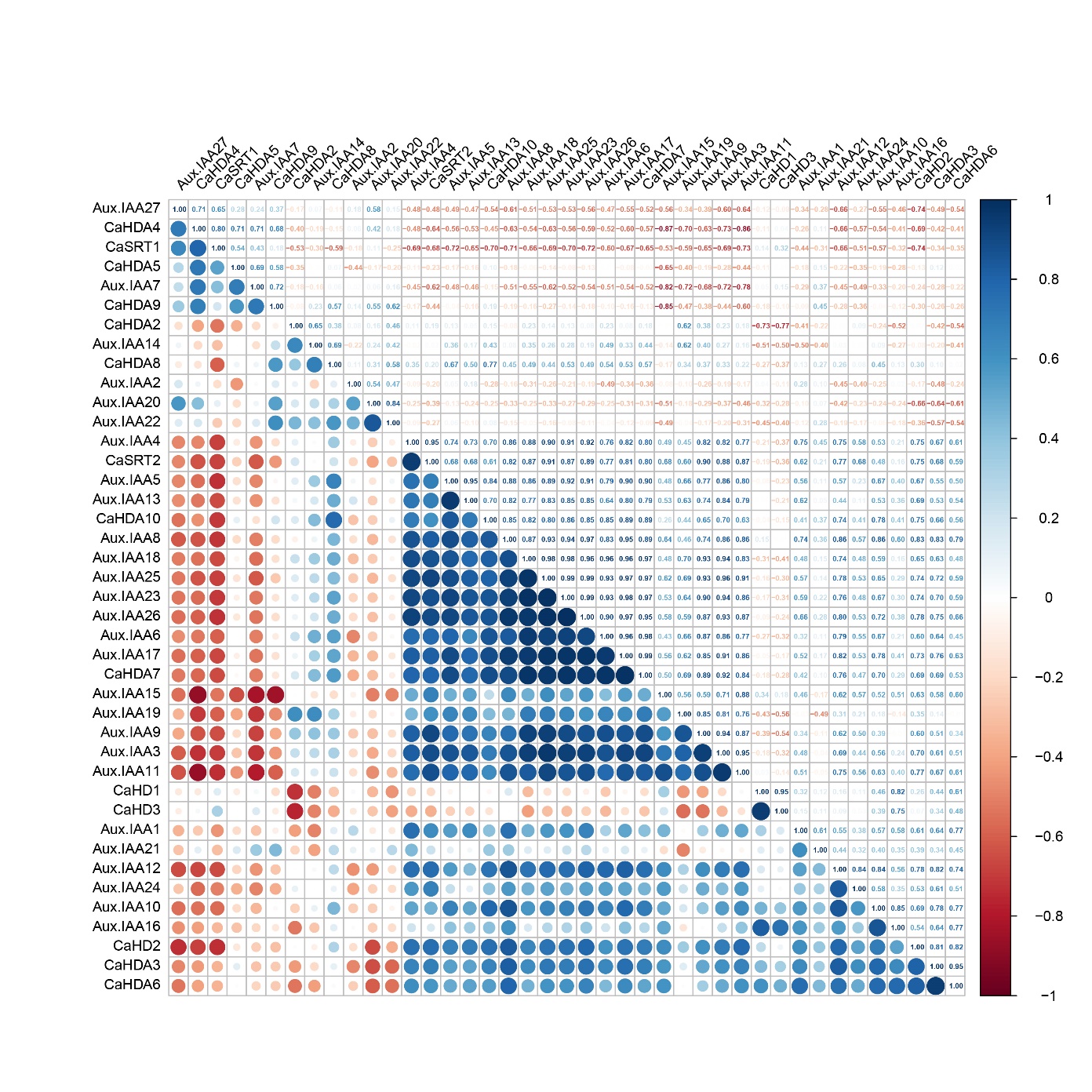


Supplementary Fig. 5 Pearson’s correlation coefficients for the *CaHDAC* and *CaAux/IAA* genes’ transcription level. The expression data was retieved from ‘Zunla-1’ transcriptome data of nine fruit developmental stages.


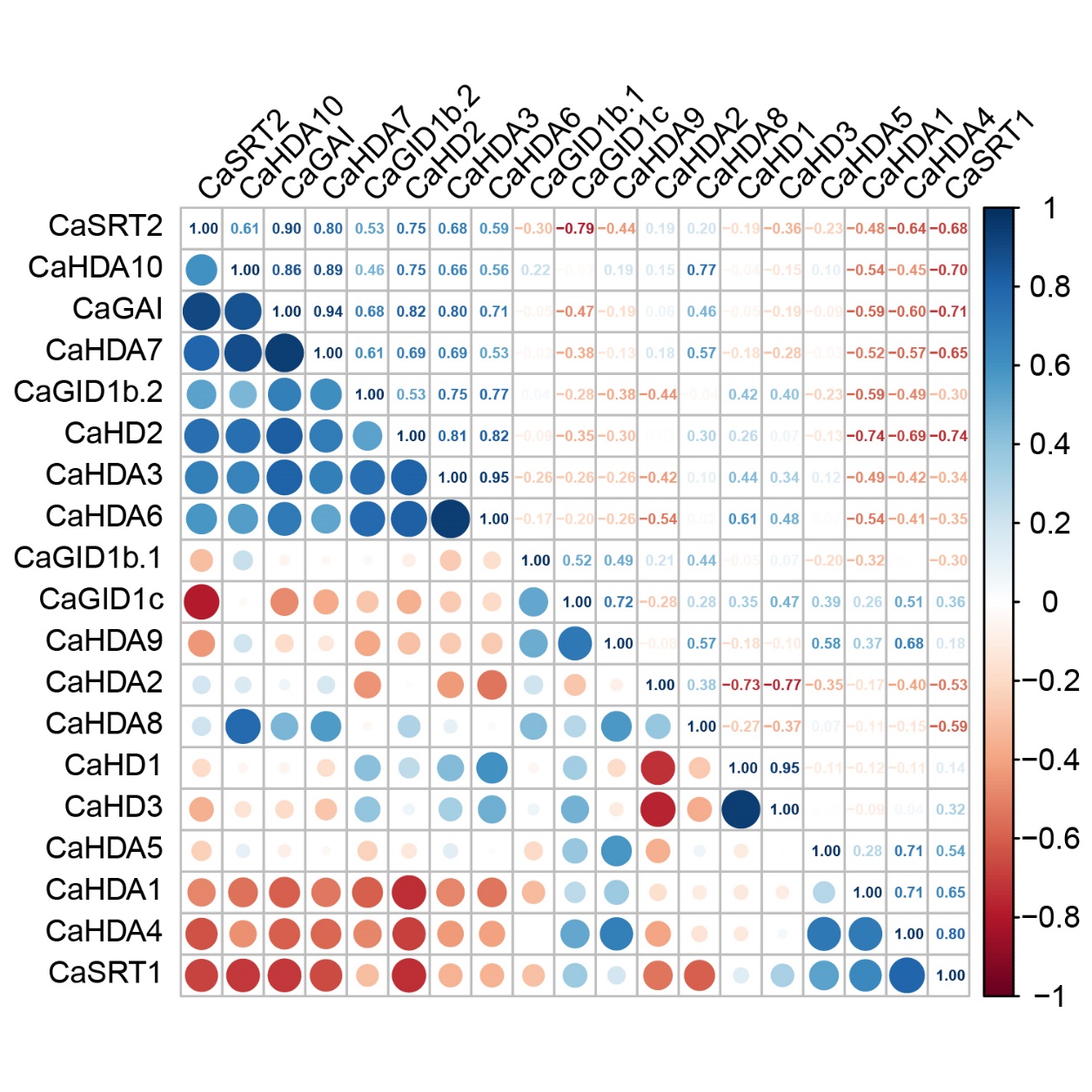


Supplementary Fig. 6 Pearson’s correlation coefficients for the CaHDAC and GA signaling genes’ transcription level. The expression data were retrieved from ‘Zunla-1’ transcriptome data of nine fruit developmental stages.


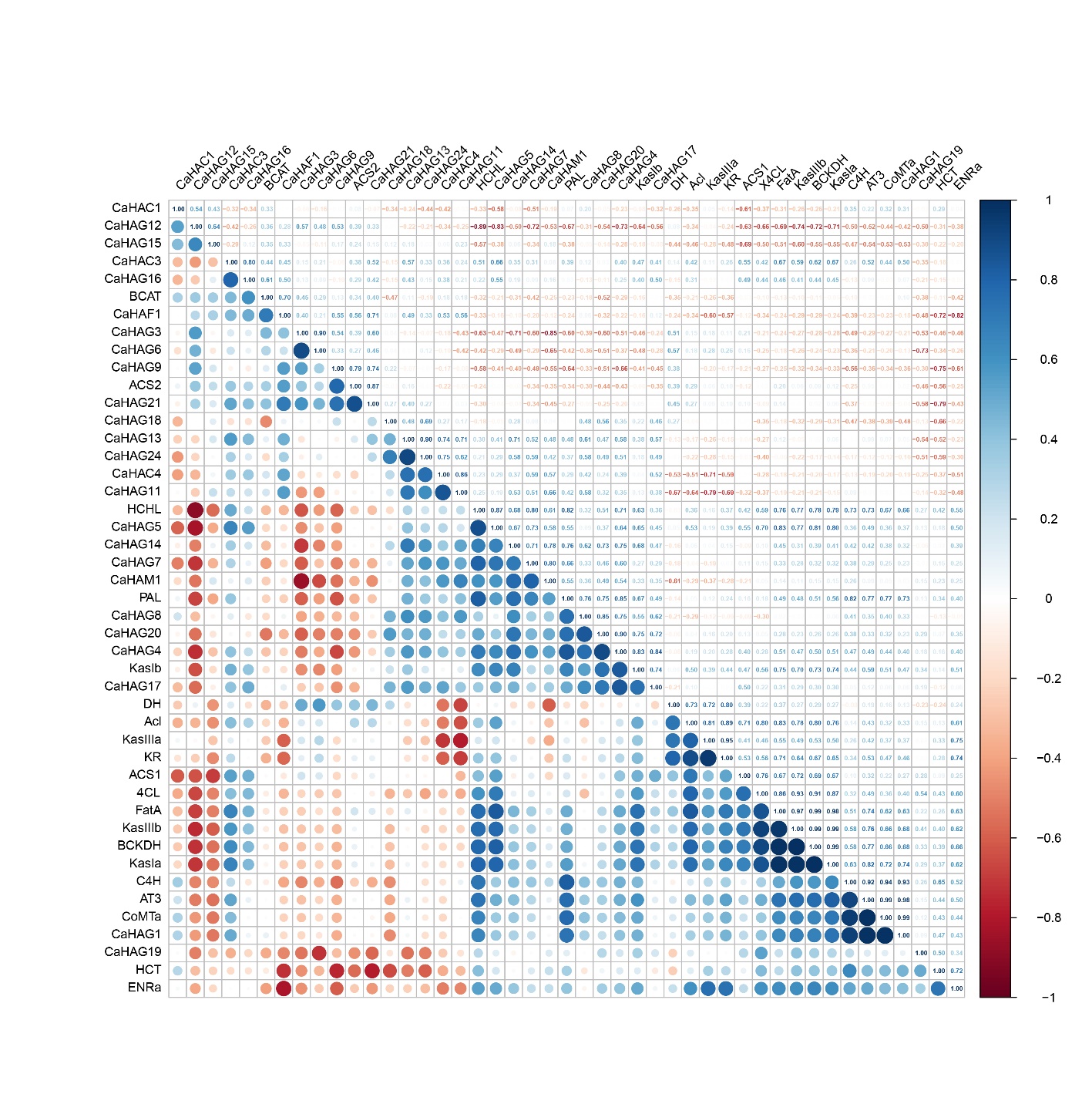


Supplementary Fig. 7 Pearson’s correlation coefficients for the CaHAT and capsaicinoid regulatory and biosynthetic genes’ transcription level. The expression data were retrieved from ‘Zunla-1’ transcriptome data of nine fruit developmental stages.


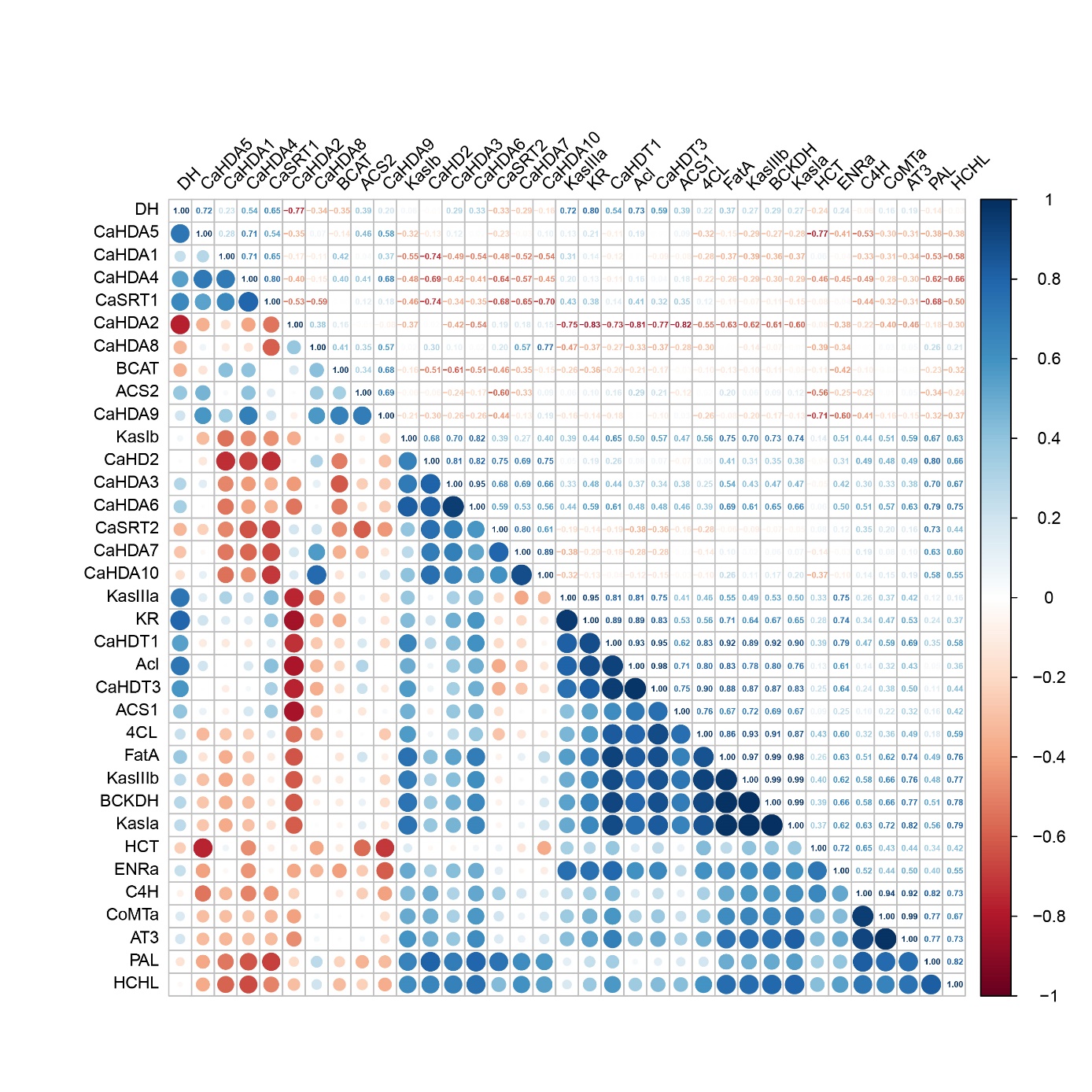


Supplementary Fig. 8 Pearson’s correlation coefficients for the CaHDAC and capsaicinoid regulatory and biosynthetic genes’ transcription level. The expression data was retieved from ‘Zunla-1’ transcriptome data of nine fruit developmental stages.
